# CaPri: Bayesian Mendelian randomization for joint estimation of causal and pleiotropic effects

**DOI:** 10.64898/2026.10.13.26365454

**Published:** 2026-10-14

**Authors:** Ou Ren, Wenmin Zhang, Tianyuan Lu

## Abstract

**Motivation:**

Horizontal pleiotropy remains a major challenge for Mendelian randomization (MR), particularly as increasingly large genome-wide association studies identify many genetic instruments for complex traits. Existing pleiotropy-robust methods rely on assumptions regarding the proportion or distribution of valid instruments that may be violated when pleiotropy is widespread.

**Results:**

We developed CaPri, a Bayesian MR framework that jointly estimates causal and pleiotropic effects. CaPri allows a potentially arbitrary proportion of instruments to contribute to a shared directional pleiotropic component and estimates the posterior probability and magnitude of the causal effect, instrument-specific posterior inclusion probabilities for pleiotropy, and the shared pleiotropic effect. Across simulations, CaPri maintained appropriate false positive control and accurate causal effect estimation regardless of the proportion of pleiotropic instruments, while accurately identifying pleiotropic instruments and estimating their shared effect. Performance remained robust across varying causal and pleiotropic effect sizes and exposure-outcome confounding, although sufficiently large outcome GWAS sample sizes were important for stable inference and limited prior dependence. In real data analyses, CaPri reported low posterior probabilities of causality across negative controls and strong support for established positive control relationships. Application to alcohol consumption and coronary artery disease further demonstrated its ability to identify potentially meaningful pleiotropic instruments. CaPri provides a complementary framework for strengthening causal inference and triangulation across MR methods with different identifying assumptions.

**Availability and implementation:**

CaPri is available at https://github.com/OuRen0104/CaPri .

